# Gain and loss events in the evolution of the apolipoprotein family in vertebrata

**DOI:** 10.1186/s12862-019-1519-8

**Published:** 2019-11-13

**Authors:** Jia-Qian Liu, Wen-Xing Li, Jun-Juan Zheng, Qing-Nan Tian, Jing-Fei Huang, Shao-Xing Dai

**Affiliations:** 1 School of Life Sciences, Zhengzhou University, Zhengzhou, 450001 China; 2 Key Laboratory of Animal Models and Human Disease Mechanisms, Kunming Institute of Zoology, Chinese Academy of Sciences, Kunming, 650223 China; 3 Kunming College of Life Science, University of Chinese Academy of Sciences, Kunming, 650204 China

**Keywords:** Phylogenesis, Gain and loss events, Divergence, Apolipoprotein, Vertebrata

## Abstract

**Background:**

Various apolipoproteins widely distributed among vertebrata play key roles in lipid metabolism and have a direct correlation with human diseases as diagnostic markers. However, the evolutionary progress of apolipoproteins in species remains unclear. Nine human apolipoproteins and well-annotated genome data of 30 species were used to identify 210 apolipoprotein family members distributed among species from fish to humans. Our study focused on the evolution of nine exchangeable apolipoproteins (ApoA-I/II/IV/V, ApoC-I~IV and ApoE) from *Chondrichthyes*, *Holostei*, *Teleostei*, *Amphibia*, *Sauria (*including *Aves)*, *Prototheria*, *Marsupialia* and *Eutheria*.

**Results:**

In this study, we reported the overall distribution and the frequent gain and loss evolutionary events of apolipoprotein family members in vertebrata. Phylogenetic trees of orthologous apolipoproteins indicated evident divergence between species evolution and apolipoprotein phylogeny. Successive gain and loss events were found by evaluating the presence and absence of apolipoproteins in the context of species evolution. For example, only ApoA-I and ApoA-IV occurred in cartilaginous fish as ancient apolipoproteins. ApoA-II, ApoE, and ApoC-I/ApoC-II were found in *Holostei, Coelacanthiformes*, and *Teleostei,* respectively, but the latter three apolipoproteins were absent from *Aves*. ApoC-I was also absent from *Cetartiodactyla*. The apolipoprotein ApoC-III emerged in terrestrial animals, and ApoC-IV first arose in *Eutheria*. The results indicate that the order of the emergence of apolipoproteins is most likely ApoA-I/ApoA-IV, ApoE, ApoA-II, ApoC-I/ApoC-II, ApoA-V, ApoC-III, and ApoC-IV.

**Conclusions:**

This study reveals not only the phylogeny of apolipoprotein family members in species from *Chondrichthyes* to *Eutheria* but also the occurrence and origin of new apolipoproteins. The broad perspective of gain and loss events and the evolutionary scenario of apolipoproteins across vertebrata provide a significant reference for the research of apolipoprotein function and related diseases.

## Background

Apolipoproteins are components of plasma lipoproteins and are mainly synthesized in the small intestine and liver. The major human apolipoproteins include ApoA, ApoB, ApoC, and ApoE. ApoA-I and ApoA-II are the major proteins of plasma high-density lipoproteins (HDLs); both ApoA-I and ApoA-IV are found in chylomicrons and HDLs, while a large portion of ApoA-IV is lipid free [1–3]. Human ApoA-V is present at a very low concentration in plasma and as a fraction of very low-density lipoproteins (VLDLs), HDLs and chylomicrons [4, 5]. ApoCs are constituents of chylomicrons, VLDLs, and HDLs [6], and ApoE is a component of various lipoprotein particles, including VLDLs and their components, chylomicrons and their components and HDLs [7], in humans.

As a crucial structural component of lipoproteins, apolipoproteins form different kinds of lipoproteins and play significant roles in lipid transport and lipoprotein metabolism [6, 8–12]. Abnormalities in apolipoproteins are a risk factor for a variety of human diseases. For example, a deficiency in ApoC-II and 24 genetic mutations can cause hypertriglyceridemia (HTG) [13]. The level of ApoC-III in human plasma is elevated in hyperlipidemia and diabetes [14, 15]. Low-density lipoprotein (LDL) accumulation in plasma resulting from a deficiency in LDL receptors (ApoB and ApoE) is related to the onset of accelerated coronary artery disease [16]. In addition, ApoE gene allelic variants [17, 18] and single nucleotide polymorphisms (SNPs) in different regions [19], together with protein level, are associated with the risk of Alzheimer’s disease [20–24].

The human exchangeable apolipoproteins APOA/C/E (ApoA, ApoC, and ApoE) have the same genomic structure and are members of a multigene family. In humans, apolipoprotein genes have similar structures, including 3′- and 5′-untranslated regions, 3–4 introns, and 2–3 exons. The exons encode signal peptides, propeptides and mature peptides. Apolipoproteins have a common 33-codon block and variable repeats in the mature peptide region. Thus, their protein domains consist of one 33-amino acid repeat and various 22- and 11-amino acid repeats. Genes for human apolipoproteins belonging to the apolipoprotein A1/A4/E family are located on three different chromosomes. In *Homo sapiens*, the *APOE/C1/C2/C4* gene cluster is located on chromosome 19q13.32. The gene *APOC1* (6.6 kb), together with its downstream pseudogene, the *APOC1’* (6.0 kb*)* gene, is located in the downstream gene *APOE* (4.7 kb) [25]. The gene *APOC2* (4.7 kb) is located between the gene *APOC1* and gene *APOC4* (4.2 kb) [26–28]. Another gene cluster of human apolipoproteins is the *APOA1/APOC3/APOA4* cluster on chromosome 11q23.3, which spans a range of approximately 43.8 kb [29, 30]. The gene *APOC3* (4.1 kb) is located upstream of *APOA4* and 4735 bp downstream of the *APOA*1 (2.9 kb) gene. The *APOA5* gene (4.0 kb) is located 31.4 kb downstream of the *APOA4* gene (3.4 kb). Only the *APOA2* (1.7 kb) gene is located on chromosome 1q23.3.

A previous study showed that the apolipoprotein LAL1 (lamprey apolipoprotein 1) in *Petromyzon marinus* (lamprey) is similar to human ApoA-I/II/IV, ApoE and ApoC-III [31]. This suggests that the APOA/C/E family and LAL1 likely share common ancestors. Members of the mouse apolipoprotein family are highly conserved with members of the human apolipoprotein family [32, 33]. The apolipoprotein ApoA genes of primates and hedgehogs independently underwent convergent evolution through different duplication and modification events [34, 35]. Another study showed that the evolution of ApoE is highly influenced by feeding habits, and ApoE of frogs as an ancestor is less related to ApoE of other species [36].

As early as 1988, Wen-Hsiung Li et al. summarized the knowledge of the biosynthesis, structure, and structure-function relationships of the APOA/C/E family and proposed a hypothetical scheme for the evolution of this family [27]. Since then, numerous studies have explored the evolution of the APOA/C/E family in different aspects and specific lineages. However, systematic comparisons and analyses of all members of the APOA/C/E family in various species throughout vertebrata are still lacking. The determination of the evolution of each apolipoprotein in vertebrata is important for understanding the implication of the function of the APOA/C/E family and the adaptation of specific species. In recent years, a large amount of genomic data has become available and provides a favorable opportunity to study apolipoproteins in a broad perspective. In this study, we systematically analyzed all members of the APOA/C/E family across vertebrata throughout *Chondrichthyes, Holostei, Teleostei, Amphibia, Sauria (including Aves)* and *Mammalia* via a comparative genomic approach. The analysis revealed the evolutionary relationships and the gain and loss events for apolipoprotein family members ApoA (I, II, IV, V), ApoC (I, II, III, IV), and ApoE and uncovered the connection between the evolution of apolipoprotein family members and their biological function in the process of species formation.

## Results

### Overall distribution of the apolipoprotein family in vertebrata

**Fig. 1:**
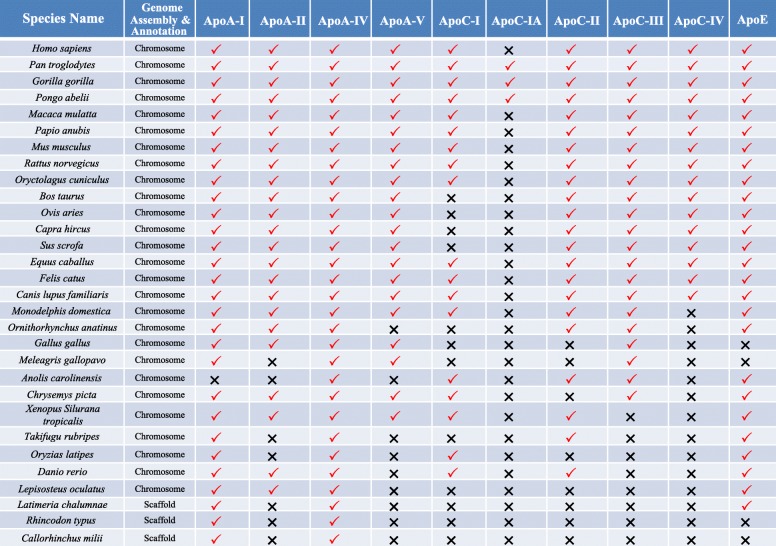
The overall distribution of the apolipoprotein family in vertebrata. A total of 10 members of the apolipoprotein family were analyzed in 30 species. The genome assembly and annotation of the top 26 species are at the chromosome level, and the other 4 species are at the scaffold level. The red check and the black ‘x’ indicate the presence and absence of an apolipoprotein in a specific species, respectively

After databases were searched and data were filtered, we obtained all genome data and protein sequences of apolipoproteins for 30 species across vertebrata (Fig. 1). The following analysis is based on the data of these 30 species. After redundant and partial sequences were eliminated, we identified 210 protein sequences belonging to ApoA (I, II, IV, V), ApoC (I~IV), and ApoE in these species by BLASTP as described in the Methods section. The overall distribution of the nine apolipoproteins in vertebrata was greatly varied. The apolipoproteins ApoA-I and ApoA-IV are present in all 30 species. The apolipoprotein ApoC-III exists extensively in *Amniota*. However, ApoC-I, ApoC-II and ApoE are absent in *Aves*. The apolipoproteins ApoC-I and ApoC-IA are not widely distributed and are present only in specific clades. For example, ApoC-IA is identified only in nonhuman *Primates*.

### Divergence and convergence between species evolution and apolipoprotein phylogeny

**Fig. 2:**
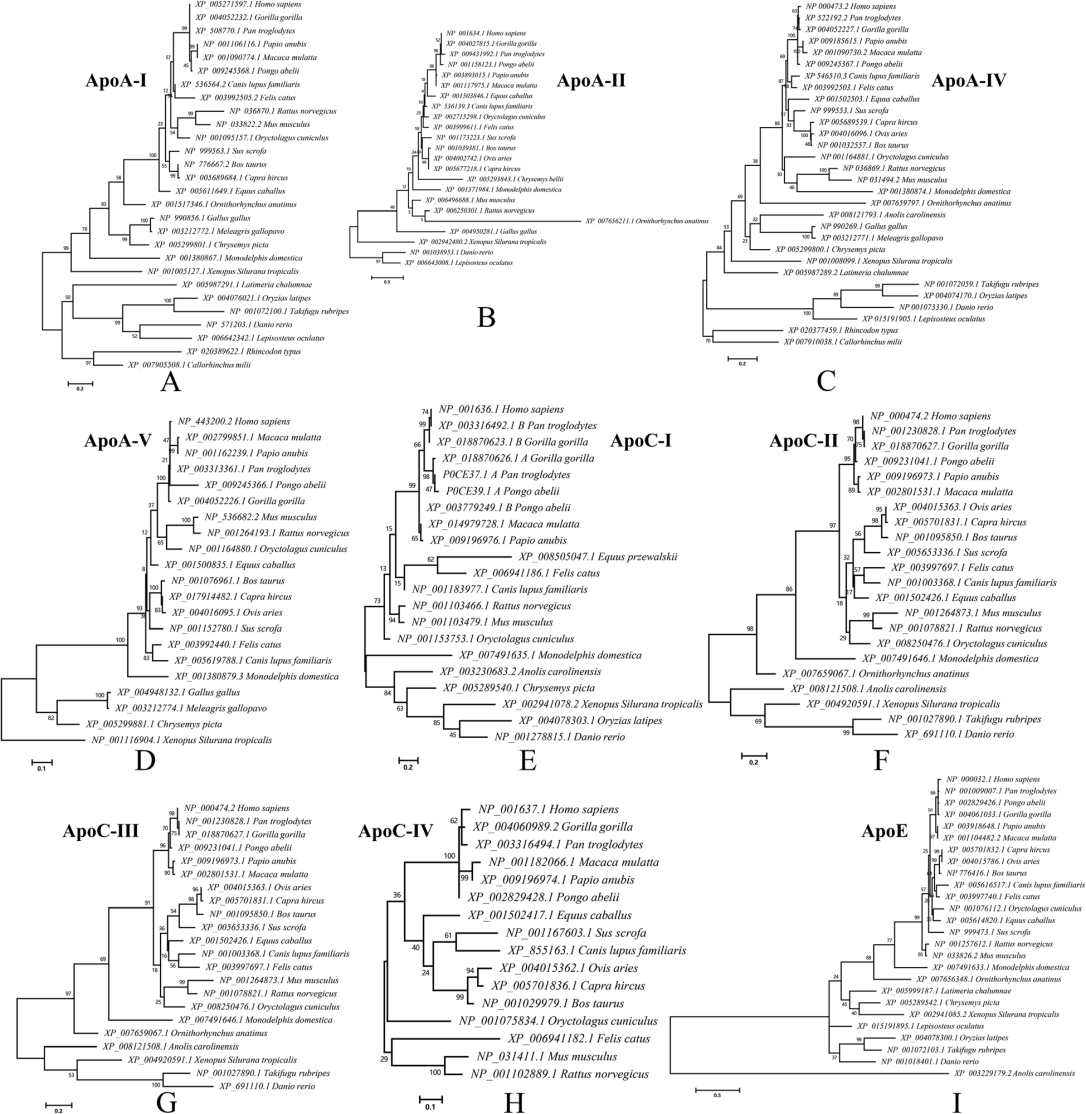
Molecular phylogenetic analyses of ApoA-I/II/IV/V, ApoC-I~IV and ApoE by the maximum-likelihood method. **a**~**i** represents the evolutionary history of ApoA-I/II/IV/V, ApoC-I~IV and ApoE, respectively. The database ID of each sequence is shown. There were a total of 45/66/212/207, 53/90/41/93 and 133 positions for these trees, respectively. The evolutionary history analyses were processed by using the maximum-likelihood method in MEGA7 as mentioned above

During the process of species evolution, the gene duplication of apolipoproteins resulted in many orthologous and paralogous genes. For each apolipoprotein family member, we gathered the sequences of its orthologous genes to construct phylogenic trees. According to the trees (Fig. 2), each apolipoprotein generally clustered into a different clade (*Chondrichthyes, Holostei, Teleostei, Amphibia, Sauria*, *Prototheria* (*Ornithorhynchus*), *Metatheria* (*Monodelphis*), *Laurasiatheria* (*Perissodactyla*, *Cetartiodactyla,* and *Carnivora*), and *Euarchontoglires* (*Primates* and *Glires*)), which is consistent with the pattern of species evolution. However, some apolipoproteins had phylogenic divergences with the evolutionary progress of species.

In the maximum-likelihood (ML) tree of the ApoA-II protein sequences, the *Glires* clade clustered with *Eutheria* (even with a very low bootstrap value) (Fig. 2b) rather than clustering with *Primates* in the *Euarchontoglires* clade. Except for ApoA-I (Fig. 2a), *Glires* consistently clustered as a peripheral clade instead of clustering with the *Primates* clade. Moreover, in ApoE ML trees (Fig. 2i), *Oryctolagus cuniculus* (rabbit) had low homology with *Mus* and *Rattus*, although rabbit and mouse both belong to *Glires*. The special evolutionary status of the *Glires* apolipoprotein may reflect its functional divergence from its orthologous proteins.

In addition to the unique *Glires* clade, apolipoprotein in platypus (*Ornithorhynchus*) and opossum (*Monodelphis*), which evolved as early mammals, also had phylogenic divergences compared with species evolution. For example, in the ApoA-I ML tree (Fig. 2a), opossum clustered at the periphery of *Sauria*.

In addition, it is noteworthy that the branch length of different clades in the trees of different apolipoproteins varied greatly. This suggests that the evolutionary rate of this family varied depending on species specificity and living environment. For example, the branch length of the *Eutheria* clade is shorter than that of the other branches in the ApoA-I and ApoE trees.

### Frequent loss of members in the evolution of the apolipoprotein family in vertebrata

**Fig. 3:**
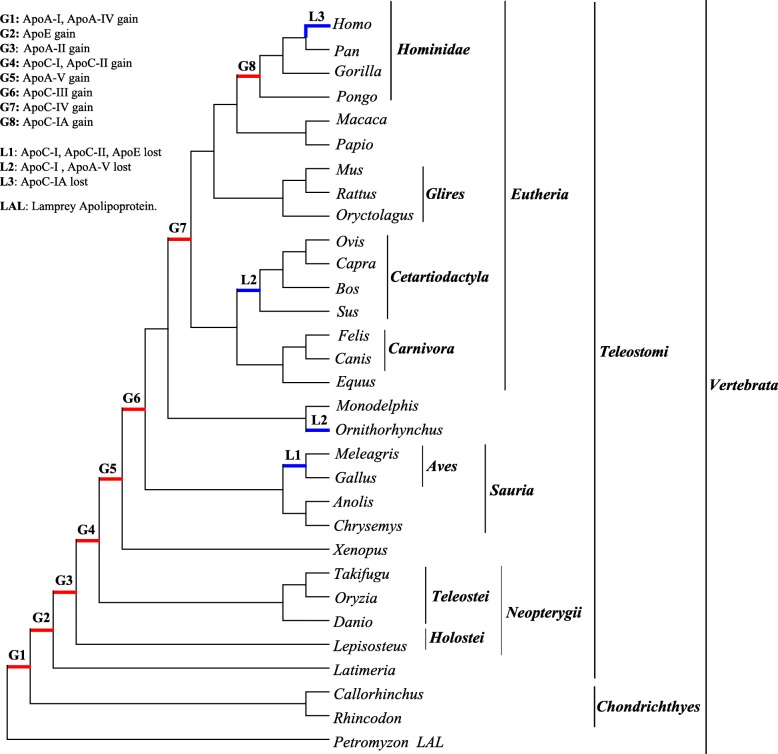
The overall evolutionary process of apolipoproteins. As the topology of the species phylogenetic tree shows, apolipoprotein family members were gained and lost in certain species during species evolution. The red and blue lines demonstrate evolutionary gain and loss events, respectively, as well as the letters ‘G’ and ‘L’. Letters and numbers above the line represent different evolutionary events that have been listed below. LAL represents lamprey apolipoprotein 1

To study the gain and loss events in the evolution of apolipoproteins, we evaluated the presence and absence of these proteins for each species (Fig. 1) in the context of species evolution. Although the functions of these proteins are conserved and, importantly, were revealed in a previous study, we found multiple gain and loss events in the evolution of apolipoproteins (Fig. 3). As indicated by the marks on the tree, there are eight gain events of apolipoproteins. The earliest members that appeared in *Chondrichthyes* (shark) were ApoA-I and ApoA-IV, and ApoE and ApoA-II were originally present in the ancient fish lineages spot gar and coelacanth, respectively. Then, ApoC-I and ApoC-II emerged in *Teleostei*. Subsequently, ApoA-V, ApoC-III and ApoC-IV emerged in *Amphibia*, *Sauria* and *Eutheria*, respectively. In addition, ApoC-I derived its acidic form, ApoC-IA, distinctly from the original basic form in *Hominidae*. However, at the branch of *Homo*, ApoC-IA was lost, and the coding gene of ApoC-IA became a pseudogene.

During species evolution, ApoC-I, ApoC-II, and ApoE were lost in *Aves* (L1 event); subsequently, ApoC-I was lost in *Cetartiodactyla* (L2 event). ApoA-V appears in *Amphibia* but disappears together with ApoC-I in platypus (*Ornithorhynchus*). Although ApoC-III and ApoC-IV appeared in *Sauria* and *Eutheria,* respectively (G3 and G4 event), and later than other apolipoproteins, they remain stable in the process of species evolution. These apolipoproteins emerged in diverse speciation events and were lost at different times. This result means that apolipoprotein family members did not all simultaneously occur at the beginning of the species and were not always preserved during evolution.

### Evolutionary relationships among the apolipoproteins ApoA, ApoC and ApoE

**Fig. 4:**
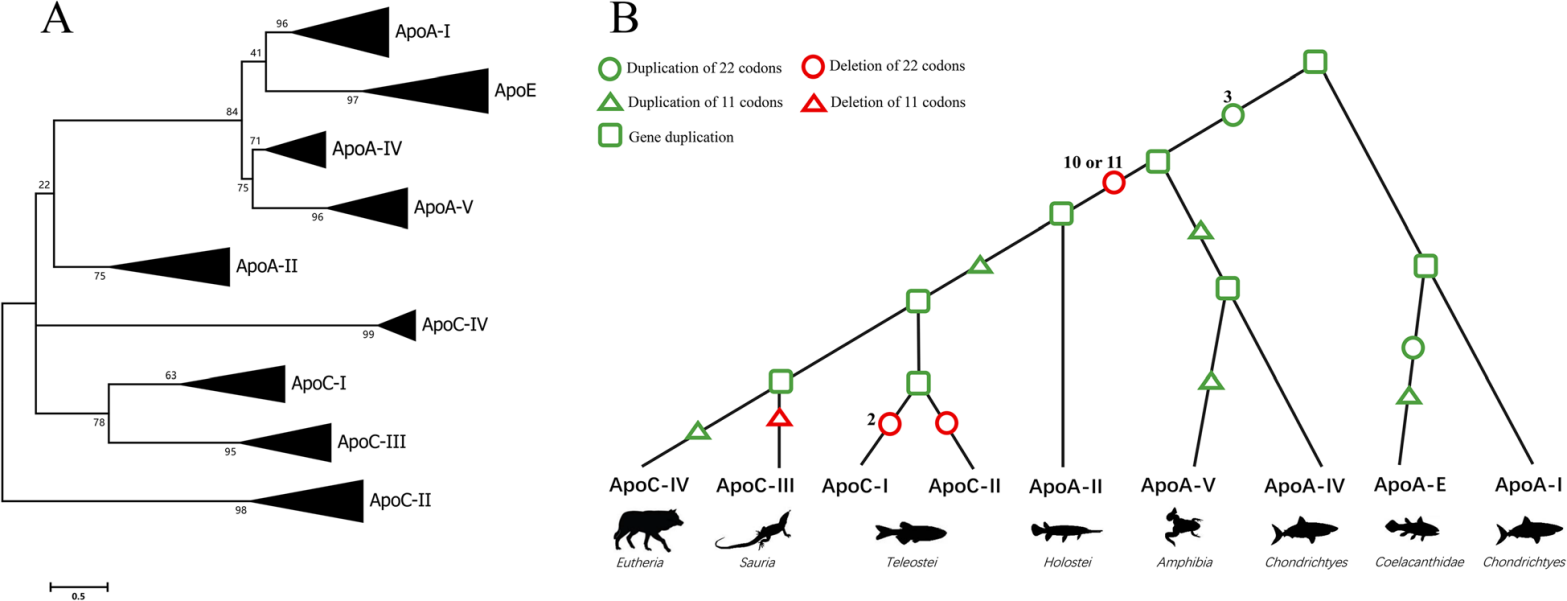
The hypothetical evolutionary scenario of apolipoproteins across vertebrates. **a**. Molecular phylogenetic analysis of nine apolipoproteins. The analysis involved the amino acid sequence of each apolipoprotein member from 30 species, and there were a total of 406 positions in the final dataset. Each branch is marked by its apolipoprotein name on the right side. **b.** A hypothetical diagram for evolutionary events of apolipoprotein genes. Gene duplication and deletion events are shown by green and red shapes, and the taxon that gained each new gene is displayed above

A comprehensive ApoC/C/E unrooted phylogenetic tree including 210 sequences from 30 species was constructed and analyzed (Fig. 4a). As shown in the tree, each branch consists of sequences from all species in which the apolipoprotein exists and clusters well together. The tree also indicates that ApoA-I, ApoE, ApoA-IV and ApoA-V cluster into one clade with high bootstrap values. Moreover, the former two and the latter two branches cluster with each other. The other members, ApoC-I~IV and ApoA-II, cluster as periphery clades, and ApoC-I is clustered closer to ApoC-III than other apolipoproteins. The phylogenetic tree reflects the relationship of apolipoproteins at the protein level.

The ApoA-I, A-II, A-V, ApoC-I~III and ApoE genes have three introns separating four exons, but the ApoA-IV and ApoC-IV genes have two introns and three exons. These genes also partly share similar repeat patterns and have a common block of 33 codons in the third exon. According to these structural characteristics, an evolutionary schematic diagram of apolipoproteins at the gene level has been proposed (Fig. 4b). The structure and length of the ancestral apolipoprotein gene are similar to ApoA-I or ApoA-IV; this primordial gene duplicated in *Chondrichthyes*, with one lineage becoming ApoA-I and one lineage becoming ApoA-IV after losing the first intron, gaining three duplications of 22 codons and one duplication of 11 codons in the fourth exon. Furthermore, in the ApoA-I lineage, the fourth exon obtained one 22-codon duplication and one 11-codon duplication, and then the duplicated gene became the ApoE gene in *Coelacanthidae*. In the ApoA-IV lineage, the gene duplicated into ApoA-V with one 11-codon duplication when *Amphibia* arose. In the latter lineage, substantial changes took place in gene length and gene duplication, and ten or eleven deletions of 11 codons occurred in the fourth exon.

Following duplication, one lineage became ApoA-II in *Holostei*, and the other became the common ancestor of ApoC. In the latter lineage, the duplication of 11 codons in the fourth exon occurred in *Teleostei*, and then the gene was further duplicated into two genes. These two resultant genes deleted one and two 22-codon repeats, leading to ApoC-II and ApoC-I, respectively. In the other lineage, the gene duplicated, with the deletion of 11 codons in the gene in *Sauria* and with the duplication of 11 codons in the gene in *Eutheria,* and became the present ApoC-III and ApoC-IV genes, respectively.

## Discussion

Apolipoprotein family members are constantly amplified and changed with the evolution of species and perform important physiological functions in these species. Although Wen-Hsiung Li et al. summarized the structural features and evolution of apolipoprotein in the 1980s, their study was limited to three species (humans, dogs and mice) and few apolipoproteins [29, 31–33]. Because of the lack of genome data for many species at that time, the conclusions of those studies are limited and cannot be applied to all vertebrates. In this research, we presented new insights that are very different from those of previous studies by the integrated analysis of a large amount of genome data of all vertebrates. We collected all ApoA, ApoC, and ApoE family members (ApoA-I/II/IV/V, ApoC-I~IV and ApoE) from *Chondrichthyes*, *Holostei*, *Teleostei*, *Amphibia*, *Sauria* (including *Aves*), *Prototheria* (*Ornithorhynchus*), *Metatheria* (*Monodelphis*), *Laurasiatheria* (*Perissodactyla*, *Cetartiodactyla*, and *Carnivora*), *and Euarchontoglires* (*Glires* and *Primates*). Representative species can indicate the dynamic changes in apolipoprotein family members at various stages and nodes of a vertebrate phylogeny more comprehensively.

These results suggest that the ancestral members of the apolipoprotein are most likely ApoA-I and/or ApoA-IV, and other members emerged subsequently after gene duplication. The order of emergence is roughly ApoA-I/ApoA-IV—ApoE—ApoA-II—ApoC-I/ApoC-II—ApoA-V—ApoC-III—ApoC-IV. This new discovery is quite different from the previous hypotheses of Wen-Hsiung Li et al., who suggested that the evolutionary change in apolipoprotein structure and length was from less structured to more structured and from short to long, respectively, by analyzing only the structural characteristics of human apolipoproteins. However, according to our study, the oldest members of the apolipoprotein family are ApoA-I and ApoA-IV, which appeared in ancient cartilaginous fish and have long amino acid sequences and complex structural compositions.

In the context of the divergence and consistency between species evolution and apolipoprotein phylogeny, almost nine apolipoproteins exhibit the phenomenon in which the clusters of protein sequences from different species are inconsistent with the evolutionary progress of these species. The *Glires* clade of apolipoproteins is usually separate from *Euarchontoglires or Primates* and does not cluster well in *Eutheria*. Indeed, through a literature search and analysis, we found that the divergent species in the genera *Mus* and *Rattus* are quite different from other species of *Eutheria* in their life history [37, 38]. In terms of body size, reproduction capacity and longevity, *Mus* and *Rattus* species differ significantly from other species. Compared with other species of *Eutheria*, species of *Mus* and *Rattus* are one-fiftieth in size, exhibit 2 to 3 times the frequency of reproduction, and have one-tenth of the average life-span; their peculiar life history inevitably has resulted in their unique mechanisms of growth and metabolism. Thus, we speculate that this diversity may lead to a marked distinction in the metabolism and transport function of lipids between *Glires* and *Primates*. This finding may explain the main reason for the effectiveness of drugs related to lipid metabolism in mouse or rat models but the ineffectiveness of such drugs in humans.

The functions of apolipoproteins not only include transferring lipids, regulating lipoprotein metabolism, and acting as receptor ligands and enzyme cofactors but also involves immune responses related to the pathogenicity factor lipopolysaccharide (LPS) [39, 40]. ApoA-I and ApoA-II, which are present in *Teleostei*, also have a crucial function in antibiosis [41, 42]. ApoC-I and ApoE are differentially expressed after bacterial infection in fish [43], and ApoA-IV is associated with food intake in zebrafish [44]. In addition, the apolipoproteins Apo-II and Apo-IV have been found to be involved in estrogen regulation and egg production [45, 46], which do not exist in *Mammalia*.

These apolipoproteins, which are primitively present in *Chondrichthyes*, have important physiological roles, and their functions have also been preserved in some species during evolution. However, our results also show that ApoC-I, ApoC-II and ApoE are absent or were lost from *Aves*. Thus, the reserved exchangeable apolipoproteins may be responsible for the lipid metabolism and fat storage needed for migration. For example, ApoA-I can bind cholesterol, high-density lipoprotein (HDL) particle receptors and phospholipids and participate in the reverse transport of cholesterol from tissues to the liver by promoting cholesterol efflux from tissues and by acting as a cofactor for the lecithin cholesterol acyltransferase (LCAT). ApoA-IV can also bind copper ions and has protein-homodimerization activity. ApoA-V can bind heparin, lipase, low-density lipoprotein (LDL) particle receptor and phosphatidylcholine.

Furthermore, some functions of apolipoprotein are unique, and once they are lost, corresponding phenotypes will appear. For example, ApoE is related to the formation of bones and appeared in bony fish, not in cartilaginous fish [47, 48]. ApoE may provide a basis for bone structure changes in the evolution of cartilaginous fish to bony fish. However, ApoE is absent in birds and may be associated with specific phenotypes, such as hollow bones without bone marrow and slender ductile bone walls. ApoC-III is the essential component of triglyceride-rich very low-density lipoproteins (VLDLs) and HDLs in plasma. ApoC-III plays a multifaceted role in triglyceride homeostasis, promotes hepatic very low-density lipoprotein 1 (VLDL1) assembly and secretion, and attenuates the hydrolysis and clearance of triglyceride-rich lipoproteins (TRLs) [49]. Animals, from aquatic to terrestrial, consume more energy to overcome changing temperatures and living conditions. ApoC-III may promote the accumulation of triglycerides and store more energy to fight hunger, but at the same time, it is also prone to inducing obesity and cardiovascular disease when food is adequate.

It is believed that relevant evidence for the absence or presence of other members can also be found, which is also the focus and challenge of future studies.

We noticed that the sequence of apolipoprotein family members evolved from long (ApoA and ApoE) to short (ApoA-II, ApoC-I, ApoC-II, ApoC-III). Long apolipoproteins mainly play a role of lipids binding and transport. Short apolipoproteins have multiple regulatory functions such as lipase inhibitor activity (ApoA-II, ApoC-I, ApoC-III), lipase activator activity (ApoC-II), signaling receptor binding (ApoA-II), phospholipase activator activity(ApoC-II), phosphatidylcholine-sterol O-acyltransferase activator activity (ApoC-I), heat shock protein binding (ApoA-II). Therefore, based the function annotation and the sequence shortening events, we propose a hypothesis that besides the functions of lipids binding and transport, short apolipoproteins may evolve new regulatory functions and contribute to more complex and flexible lipids metabolism in vertebrate species.

## Conclusions

To summarize, we reported a lot of gain and loss events of the exchangeable apolipoproteins from *Chondrichthyes* to *Eutheria*, *which hasn’t previously been reported*. New members occurred at the nodes of speciation. Following ApoA-I and ApoA-IV, ApoE arose in Coelacanthiformes; ApoA-II arose in Holostei; ApoC-I arose in Teleostei with ApoC-II; ApoC-III and ApoC-IV emerged in Sauria and Eutheria, respectively; And ApoC-IA emerged in Hominidae. Furthermore, members also lost in the specific nodes of a vertebrate phylogeny. ApoC-I, ApoC-II and ApoE were absent from Aves; ApoC-I/ApoA-V was absent from Ornithorhynchus and Cetartiodactyla; And ApoC-IA lost in human. This is the first clarification of these gain and loss events, which will benefit the research in related fields. Additionally, we also noticed that the sequence of apolipoprotein family members evolved from long (ApoA and ApoE) to short (ApoA-II and ApoC), according to the species in which they primordially existed. Short apolipoproteins ApoA-II and ApoC may evolve new regulatory functions besides the functions of lipids binding and transport. Further data mining and functional research will focus on the function and evolution of major apolipoproteins related to human disease.

## Methods

### Collection of genome data and sequence retrieval

To study the evolution of the apolipoprotein family across vertebrata, we obtained genome information from the Ensembl database (http://www.ensembl.org/index.html). After the level of genome assembly and annotation were evaluated, only the species with the level ‘Chromosome’ were used for further analysis (Fig. 1). We downloaded the genome and protein sequences of these species. All members of the apolipoprotein family in *Homo sapiens* were retrieved from the NCBI database (https://www.ncbi.nlm.nih.gov/). These human apolipoprotein sequences (ApoA-I:NP_000030.1, ApoA-II:NP_001634.1, ApoA-IV:NP_000473.2, ApoA-V:NP_443200.2, ApoC-I: NP_001636.1, ApoC-II:NP_000474.2, ApoC-III:NP_000031.1, ApoC-IV:NP_001637.1, and ApoE:NP_000032.1) were used as query sequences against all protein sequence data using both local and online BLASTP searches under the default parameters. The details for sequence retrieval were described in our previous study [50]. To confirm the loss of genes in specific species, we first used these protein sequences to search for genome sequences by local tblastn. Then, we used these apolipoproteins to search for all available nucleotide databases in NCBI by online tblastn.

### Phylogenetic analysis of the apolipoprotein family

Phylogenetic analysis of the apolipoprotein family was performed using MEGA7 [51]. The amino acid or nucleotide sequences were multiply aligned using MUSCLE [52] with the default parameters. Alignment gaps and unmatched regions were eliminated manually. Phylogenetic trees were constructed by using the maximum-likelihood (ML) method. The ML method is based on the Jones-Taylor-Thornton (JTT) matrix-based mode or Poisson model [53]. The phylogenetic test was performed using the bootstrap method with 1000 bootstrap replications. The ML heuristic method was performed with the nearest-neighbor-interchange (NNI). Initial trees for the heuristic search were obtained automatically by applying Neighbor-Join and BioNJ algorithms to a matrix of pairwise distances estimated using a JTT model and selecting topology with superior log likelihood value. The tree was drawn to scale with branch lengths measured in the number of substitutions per site. Positions including gaps and missing data were completely deleted.

## Data Availability

The datasets generated and analyzed in the current study are publicly available on listed websites in the ‘Methods’ section.

## Abbreviations

APOA1/ApoA-I: apolipoprotein A-I
APOA2/ApoA-II: apolipoprotein A-II
APOA4/ApoA-IV: apolipoprotein A-IV
APOA5/ApoA-V: apolipoprotein A-V
ApoB: apolipoprotein B
APOC1/ApoC-I: apolipoprotein C-I
APOC1A/ApoC-IA: apolipoprotein C-IA
APOC2/ApoC-II: apolipoprotein C-II
APOC4/ApoC-IV: apolipoprotein C-IV
APOE/ApoE: apolipoprotein E
BLAST: basic local alignment search tool
BLASTP: protein-to-protein BLAST
HDL: high-density lipoprotein
JJT: Jones-Taylor-Thornton matrix
LAL1: lamprey apolipoprotein 1
LDL: low-density lipoprotein
MEGA7: molecular evolutionary genetics analysis version 7
ML: maximum-likelihood
MUSCLE: MUltiple Sequence Comparison by Log-Expectation
NCBI: National Center for Biotechnology Information
NNI: nearest-neighbor-interchange
NWM: new world monkeys
VLDL: very low-density lipoprotein
